# Driving Third-Order Optical Nonlinearities in Photoluminescent Si Nanoparticles by Nitrogen Co-Implantation in a Silica Matrix

**DOI:** 10.3390/ma15165670

**Published:** 2022-08-18

**Authors:** Jhovani Bornacelli, Fernando Arturo Araiza-Sixtos, Carlos Torres-Torres, Marco Antonio Hernández-Acosta, Alicia Oliver, Raúl Rangel-Rojo

**Affiliations:** 1 Facultad de Ciencias, Universidad Nacional Autónoma de México, Ciudad de México 04510, Mexico; 2 Departamento de Óptica, Centro de Investigación Científica y Educación Superior de Ensenada, A.P. 360, Ensenada 22860, Mexico; 3 Sección de Estudios de Posgrado e Investigación, Escuela Superior de Ingeniería Mecánica y Eléctrica Unidad Zacatenco, Instituto Politécnico Nacional, Ciudad de México 07738, Mexico; 4 Instituto de Física, Universidad Nacional Autónoma de México, Ciudad de México 04510, Mexico

**Keywords:** photoluminescence, nonlinear optics, third-order nonlinear optical effects, quantum dots, ion-implanted nanoparticles

## Abstract

The photoluminescence and third-order nonlinear optical effects of co-implanted silicon nanoparticles and nitrogen ions in a silica matrix were studied. Experimental evidence shows the potential of nitrogen ions for changing optical properties exhibited by silicon nanoparticles implanted in an integrated system. The modification of the optical bandgap and photoluminescent intensity in the studied nanomaterials by the incorporation of nitrogen was analyzed. Standard two−wave mixing experiments were conducted using nanosecond and picosecond laser pulses at 532 nm wavelength. At this off-resonance condition, only multiphoton excitation can promote electrons at energies above the optical bandgap of the silicon nanoparticles. The picosecond results show that the co-implanted sample with nitrogen exhibits a three-fold enhancement of the nonlinear Kerr response. Femtosecond z-scan measurements were undertaken at 800 nm in order to explore the modification of the ultrafast nonlinear response of the samples that revealed a purely electronic Kerr nonlinearity together to saturable absorption of the SiNPs in the near-infrared. Remarkably, femtosecond results reveal that nitrogen co-implantation in the SiNPs system derives from the quenching of the third-order nonlinear optical behavior. These findings pointed out a simple approach for engineering the optical bandgap of nanocomposites, which can be controlled by a doping process based on ion-implanted nitrogen. It is highlighted that the enhanced light-matter interactions induced by nitrogen implantation can be useful for developing nonlinear integrated silicon photonics nanodevices with low power excitation.

## 1. Introduction

Silicon photonics continues to be an important research field and is widely accepted as a key technology for next-generation communication and interconnection systems [1]. The development of silicon photonic components has opened a promising technological route to envision real applications in photosensors, optical modulators, waveguides, light sources, and all-optical switching platforms. Silicon nanostructured materials have been studied in the last decade as nano-emitters, and many strategies have been proposed and implemented to enhance their emission properties [2,3,4]. Nonlinear effects have also been studied in silicon nanomaterials at visible and infrared wavelengths, even at low excitation power, with the potential to be applied in mode-locking lasers and ultrafast optical switching [5,6,7].

Special attention has been given to the systems compound of Si nanoparticles (SiNPs) embedded in silicon dioxide or silicon nitride matrices [8]. Both matrices provide protection again external physical or chemical damage; and the methods of fabrication of this kind of system are fully compatible with CMOS technology. SiNPs are particularly attractive for future practical applications in photonic integrated circuits and all optical silicon devices. 

The scientific research on the optical properties of SiNPs in silicon nitride (SiN) films has been more extensive than the study of properties exhibited by oxide matrices such as the SiO_2_. SiN films offer lower tunneling barriers leading to better transport conditions of electrons and holes inside the SiNPs. Moreover, by varying the growth conditions, stronger light emission in a wide spectral range from UV to IR can result in SiN films [9,10]. Additionally, even though the physical mechanism that gives rise to the emission of light in SiN films has not been clarified yet, control over the Si nitride films by a doping process using nitrogen atoms by ion-implantation (which is a fully CMOS compatible technique) has been reported [11]. On the other hand, Si-rich silicon nitride has also been reported to present enhanced Kerr nonlinearity with respect to the standard stoichiometric Si_3_N_4_ materials [6,8,12,13,14]. 

With these considerations, in this work, we present the study of the optical Kerr nonlinearity exhibited by SiNPs embedded in a SiO_2_ matrix and co-implanted with nitrogen atoms. We evaluated the effect of nitrogen implantation on the absorption and photoluminescent properties of the samples. Nonlinear optical measurements were conducted to explore the Kerr nonlinearities in the samples containing N ions with ns and ps pulses at 532 nm. Additional studies of the nonlinearity are reported employing fs pulses at 800 nm in order to have a deeper characterization of the nonlinear optical response. For the experiments at 532 nm, we observed that the third-order nonlinear phenomena are enhanced in the nitrogen-implanted sample and the physical effects that give rise to these phenomena are discussed. In contrast, results at 800 nm wavelength demonstrated the potential inhibition of the third-order nonlinear optical processes induced in SiNPs by femtosecond pulses through the assistance of N ions co-implanted in the silica substrate studied. 

## 2. Materials and Methods

### 2.1. Sample Preparation

Silicon ions (Si2+) were implanted in a high purity silica matrix (Suprassil 300) with an energy of 3.5 MeV with an estimated fluence of 3 × 10^17^ ions/cm^2^ by using the 3 MV Tandem Accelerator facilities (NEC 9SDH-2 Pelletron) at the Instituto de Física (UNAM). The sample was thermally annealed at 1100 °C under a reducing atmosphere RA consisting of 50% H_2_ and 50% N_2_, for 1 h (sample labeled as SiNPs). After this initial annealing process, the sample was cut into two pieces, and one of them was implanted with nitrogen ions at 1 MeV with a fluence of 1.6 × 10^17^ ions/cm^2^. Finally, the sample implanted with N was annealed at 600 °C, also under an RA (sample labeled as SiNPs+N). According to Monte Carlo simulations performed with the SRIM program [15], the implanted Si has a depth profile ranging from 1.7 to 3.2 μm and peaked at 2.8 μm. On the other hand, the implanted N ions have a depth profile ranging from 1 to 2.2 μm and peaked at 1.7 μm. Even though the peak of the implanted ions does not coincide, these energies of implantation allow a partial overlapped of both Si and N ions depth profiles. 

### 2.2. Microscopy Characterization

Transmission electron microscopy (TEM, JEOL 2010F) observations were carried out to analyze the SiNPs embedded in the silica matrix. Before the microscopy observations took place, the sample was ground down mechanically by a Precision Ions Polishing System (PIPS) in order to expose the embedded SiNPs on a surface and to produce a film of a few hundred nanometers suitable for TEM imaging. 

### 2.3. UV-Vis Spectroscopy and Photoluminescence Studies

Optical absorption was measured with a Varian Cary Spectrophotometer (Cary 5000). Photoluminescence (PL) experiments were performed at 355 nm or 532 nm by a picosecond laser system (EKSPLA PL2143A) at 532 nm by a nanosecond laser (Continuum Surelite II-10); and also by continuous laser excitation at 488 nm (Spectra Physics Cyan 488 Externally Doubled Diode). The optical emission was acquired by a spectrophotometer (Ocean Optics USB-2000+XR1-ES) assisted by optical fibers. A convex lens with a focal length of 15 cm was employed to focus the emitted light in the fiber. The PL signals were recorded in an integration time of 1 s and filtered by a long wave-pass filter with a 90% transmittance. In order to guarantee reproducibility and explore the homogeneity of the emission in the samples studied, an XYZ mechanical stage was used to measure different regions of the implanted substrates.

### 2.4. Nanosecond and Picosecond Two−Wave Mixing Experiments

The nanosecond and picosecond third-order optical responses of the samples were separately measured by using two−wave mixing setups at 532 nm laser excitation using both picosecond (25 ps), and nanosecond (4 ns) pulses provided by Nd-YAG lasers (PL2143A 26 ps-pulsed EKSPLA system and SL II-10 ns-pulsed Continuum system; respectively) in similar geometry. 

The beam emerging at the output of each laser source was divided into pump and probe beams by using a calcite beam splitter at a rate of intensity 1:1, with linear and parallel polarizations. The incident beams were focused in approximately 0.1 mm beam diameter with a geometric angle on the incidence of about 25° between the pump and probe beams in both cases of pulse duration. The maximum incident pulse energy was 30 mJ in the nanosecond experiment and 50 μJ in the picosecond experiment. The repetition rate of the pulses was 1 Hz in both cases. A half-wave plate was employed to orient the direction of the polarization of the pump beam, while the polarization of the probe beam was fixed in the two−wave mixing measurement. Calcite polarizers were used to analyze the evolution of the polarization of the incident beams after interacting with the sample in the experiments. The optical irradiance was recorded by PIN detectors. Reproducibility of the experiments was guaranteed by studying three different regions of the sample with 10 shots to represent the average of transmitted irradiance in the same lab conditions. The error bar in acquired transmittance is around ±10%.

In order to describe the amplitudes of the electric fields through the sample in a two−wave mixing, the following wave equation was employed [16]:

(1) $$\nabla^{2}E_{\pm }=-\frac{n_{\pm }^{2}\omega^{2}}{c^{2}}E_{\pm },$$

where the optical frequency is represented by *ω* and *c* represents the speed of the light. The circular components of the right and left polarized electric fields are, respectively, written as *E*_+_ and *E*_−_. The indexes of refraction *n*_±_, for the circular components of polarization can be represented as [16]:

(2) $$n_{\pm }^{2}=n_{0}^{2}+4\pi \left(A{\left|E_{\pm }\right|}^{2}+(A+B){\left|E_{\mp }\right|}^{2}\right),$$

where the weak-field refractive index is *n*_0_. The description of *n*_±_ allows identifying different physical mechanisms responsible for the optical nonlinearity governing the transmittance given by Equation (1) with dependence on the constants *A* and *B.* These parameters are related to the third-order susceptibility tensor $\chi_{}^{\left(3\right)}$, and they take different values for an electronic phenomenon $A=\chi_{1122}^{\left(3\right)}$ and $B=\chi_{1212}^{\left(3\right)}$ while a thermal mechanism corresponds to $A=\chi_{1122}^{\left(3\right)}$ and B=0.

### 2.5. Femtosecond z-Scan Experiments

The third-order nonlinear optical properties of the nanomaterials were also explored by using the z-scan technique [17] with fs laser pulses at 800 nm provided by a Ti:sapphire oscillator. The laser employed in our studies emits 98 fs pulses with a 76 MHz repetition rate, and it has a spectral width of Δλ = 10 nm centered around 800 nm. For the z-scan technique, a 200 mm focal length lens was used to focus the irradiance *I* of the beam on the sample. The width of the beam at the focal plane was w_0_ = 28 μm. Using an aperture before a detector in the far field, we are able to study both the nonlinear absorption when the aperture is fully open (Open Aperture OA) and the nonlinear refraction when the aperture is partially open (Closed Aperture CA) when the position of the sample is scanned across the focal plane.

The two-photon absorption coefficient β involved in the irradiance-dependent absorption coefficient $\alpha \left(I\right)=\alpha_{0}+\beta I$, was considered to describe the normalized transmittance as a function of the sample position *z* in the open z-scan evaluation [17]:

(3) $$T\left(z\right)=1-\frac{\beta I_{o}L_{eff}}{2\sqrt{2}\left(x^{2}+1\right)},$$

where $x=z/z_{0}$, where *z_0_* is the Rayleigh range of the beam focused at the point $z_{0}=\pi w_{0}^{2}/\lambda$ with a spot size $w_{0}^{}$ and a wavelength λ.

The nonlinear refractive index $n_{2}$ involved in the refractive index dependent on irradiance $n=n_{0}+n_{2}I$, was related to Reχ(3) by n2=Reχ(3)/ε0n02c, and the normalized transmittance *T* resulting from the closed z-scan experiments as a function of z was estimated by [17]:

(4) $$T\left(z,\Delta \Phi_{o}\right)=1-\frac{4\Delta \Phi_{o}x}{\left(x^{2}+9\right)\left(x^{2}+1\right)},$$

the peak on-axis nonlinear phase change ΔΦ*_o_* was considered as:

(5) $$\Delta \Phi_{o}=k\Delta n_{o}L_{eff},$$

where *I*_0_ corresponds to the focused peak on-axis irradiance and $\Delta n_{0}=n_{2}I$ represents the maximum refractive index change on-axis.

### 2.6. Photothermal Evaluations

The photothermal response exhibited by the samples in the two−wave experiments was initially explored by using a FLUKE-FLKPTI120 9HZ thermographic camera and then recorded by using an electronic temperature sensor integrated by a thermistor in a Wheatstone bridge circuit. The temperature of the samples was analyzed under the same conditions of irradiation used in the two−wave mixing experiments conducted to explore the nanosecond and picosecond nonlinear optical effects.

## 3. Results

**Figure 1:**
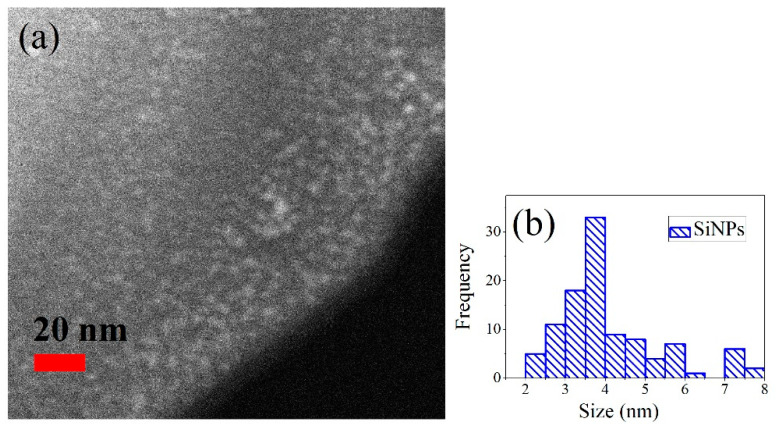
(**a**) Panoramic TEM micrograph of SiNPs embedded in SiO_2_. (**b**) Histogram showing the size distribution of the SiNPs.

**Figure 2:**
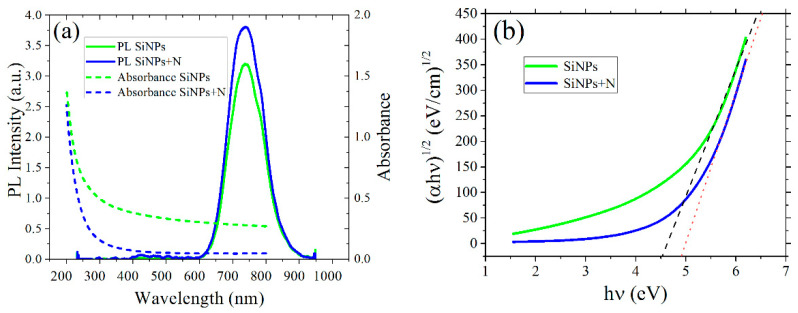
(**a**) Absorbance vs. wavelength and photoluminescence spectra for the samples studied. The excitation wavelength used in the photoluminescence experiment was 355 nm. (**b**) Tauc plot derived from the absorption curve in (**a**).

Figure 1a shows a representative TEM micrograph of the SiNPs embedded in SiO_2_. The SiNPs size distribution is shown in Figure 1b. According to the results in Figure 1, the SiNPs have a mean size of about 3–4 nm, but there is also a significant number of SiNPs with sizes between 7 and 8 nm. In general, the SiNPs size is less than 10 nm, short enough to see quantum size effects such as visible PL, as shown in Figure 2a. 

As shown in Figure 2a, the sample with SiNPs exhibited a typical PL spectrum in the red region of the visible spectrum. The PL peak for the SiNPs sample occurred at 739 nm, and it ranged from 600 nm to 900 nm. The comparison of the PL spectra for the SiNPs and SiNPs+N samples can be easily visualized in Figure 2a, the PL data have similar spectral characteristics in both cases, but the PL intensity was almost 27% stronger in the case of SiNPs+N. The optical absorption spectra for the SiNPs and SiNPs+N samples are also shown in Figure 2a, and both spectra exhibited the typical absorption edge towards the UV. Figure 2b shows the Tauc plots to find an estimation of the average bandgap of the samples. 

The interception with the energy axis shows that the effective bandgap would be near 4.5 eV and 4.9 eV for the SiNPs and SiNPs+N samples, respectively. In general, it is reported that the optical bandgap for SiNPs is between 1.3 to 3.5 eV (953 to 354 nm) for nanoparticle sizes between 1 and 8 nm [18,19,20]. The bandgap for fused silica is usually in the range of 8 to 10 eV. Then it seems that the estimated bandgap from the data shown in Figure 2b is an average of the bandgap of SiNPs and the SiO_2_ host matrix. However, the host matrix is the same for both samples, and we can deduce that the optical bandgap of the sample implanted with N is slightly higher than that measured in the sample with only SiNPs. 

**Figure 3:**
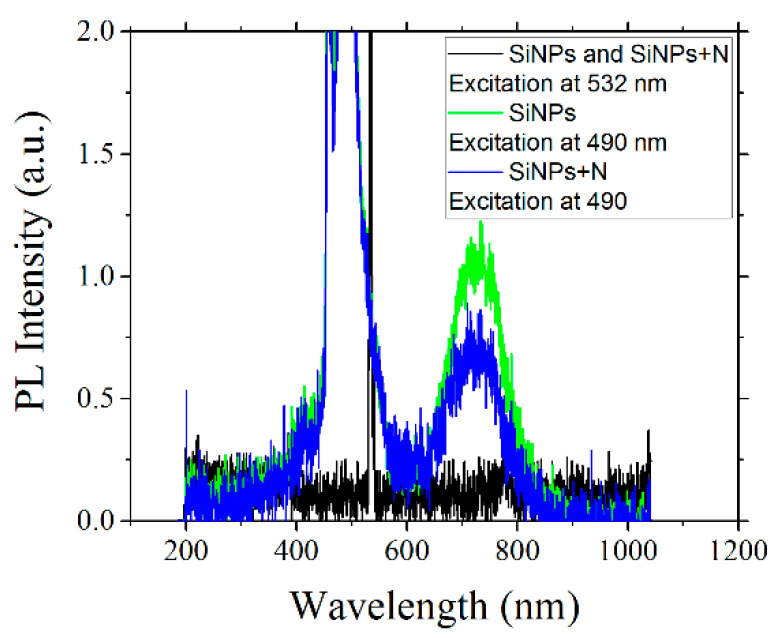
Photoluminescence spectra for the SiNPs and SiNPs+N sample by using two different excitation wavelengths: 490 nm (blue and green plots) and 532 nm (black plot).

From the absorption data plotted in Figure 2a, we can see that it is more probably to transfer an electron through the optical bandgap of SiNPs at shorter excitation wavelengths, and then the probability of PL emission increases accordingly. On the contrary, for large wavelength excitation, the PL intensity from SiNPs decreases. Eventually, at longer wavelength excitation closer to the emission wavelengths, it is not possible to have single-photon excitation of the SiNPs PL. In Figure 3, we can see the PL emission from the SiNPs and SiNPs+N samples when they are excited at laser wavelengths of 532 nm and 490 nm. This experiment clearly shows that for excitation at 490 nm, a weak PL band is observed, not visible to the naked eye, as shown in the blue and green plots in Figure 3. The PL intensity for the SiNPs+N sample is lower with respect to the emission in the SiNPs sample. It is worth mentioning that the effective optical band gap in the SiNPs+N sample is larger than in the SiNPs sample. Evidently, while further from resonance is the excitation wavelength, a decrease in the probability of the electronic optical transitions occur, and the PL emission decreases accordingly. In good agreement, excitation at 532 nm did not produce any discernable PL for both samples. We can then assume that for this 532 nm wavelength, it is not possible to promote electrons through the optical bandgap of the SiNPs, at least for a single-photon absorption process. 

**Figure 4:**
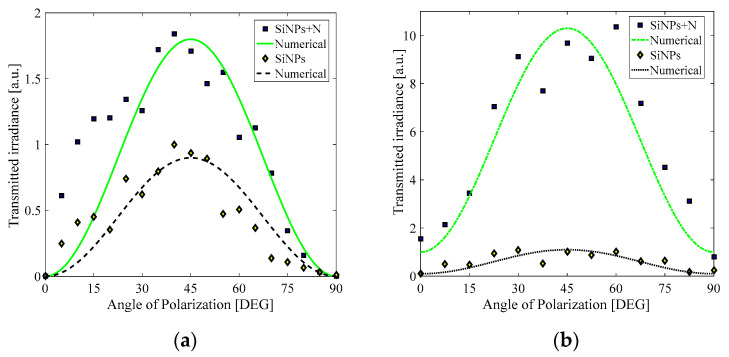
Kerr transmittance at 532 nm by (**a**) nanosecond pulses and (**b**) picosecond pulses.

However, it is possible to excite electrons from the valence to the conduction band of the SiNPs by multiphotonic absorption induced by an intense optical field generated by pulsed laser excitation. As it can be seen from the Tauc plots in Figure 2b, the optical bandgap of the sample implanted with N atoms has been modified. Then it is expected that, under pulsed laser irradiation, different nonlinear absorption coefficients will be obtained for the two samples studied. In order to prove this hypothesis, we conducted a two−wave mixing experiment in the SiNPs and SiNPs+N samples under nanosecond and picosecond laser excitation; the results are shown in Figure 4. As is evidenced from the plots in Figure 4, the Kerr transmittance is more intense in the SiNPs+N sample compared to the SiNPs one. Moreover, under picosecond laser excitation, this Kerr effect enhancement is larger than that obtained for the ns pulses. 

The effective third-order nonlinear susceptibilities estimated for each sample are presented in Table 1. For picosecond pulses, there is a 3-fold increase in the third-order susceptibility for the SiNPs+N sample with respect to the SiNPs sample.

**Figure 5:**
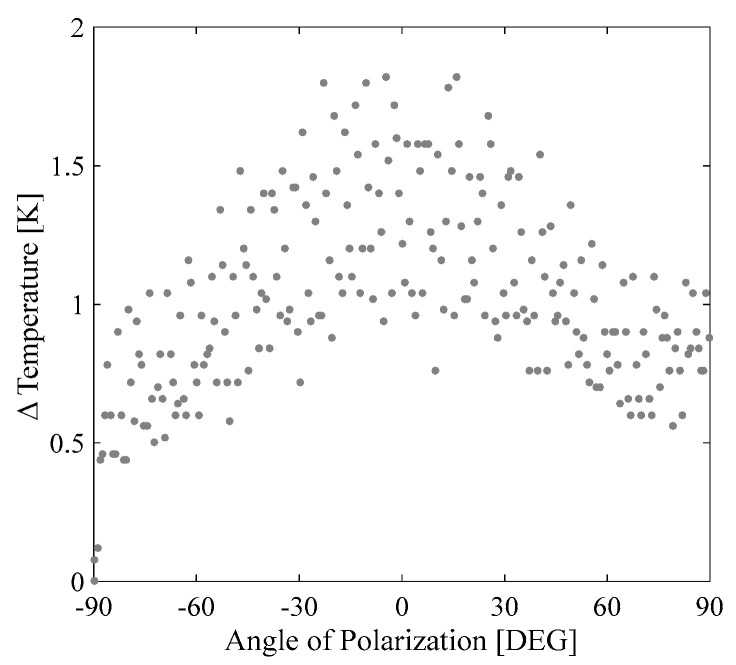
Change in temperature (ΔT) vs. angle of polarization of the incident beams in a two−wave mixing experiment in the SiNPs+N sample irradiated by nanosecond pulses at 532 nm.

In order to guarantee that different physical mechanisms were involved in the third-order optical nonlinearities exhibited by the sample SiNPs, we evaluated the photothermal effects induced by nanosecond and picosecond pulses. The absence of a change in temperature was obtained by the picosecond pulses, but a clear variation in temperature was identified by using the nanosecond pulses. The picosecond nonlinear optical response is then expected to be purely electronic. We noticed that the maximum average change in temperature of the SiNPs sample was found for parallel polarization of the incident beams; but remarkably, an inhomogeneous temperature change can be obtained by a polarization-resolved interaction of the linearly polarized beams when the thermal sensor is located in different positions in respect to the SiNPs+N sample. Figure 5 illustrates a representative case of the temperature change induced for the sensor orthogonally positioned with respect to the propagation of the beams.

**Figure 6:**
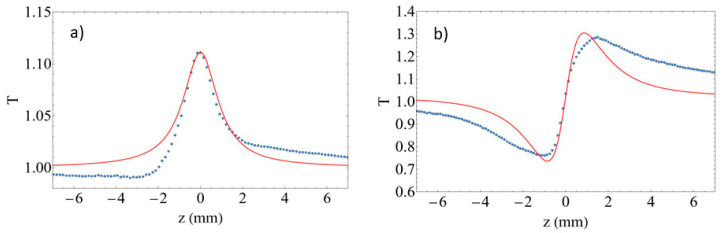
Z-scan results obtained with 98 fs pulses at 800 nm using a 300 mW average power (corresponding to a pulse energy of 4 nJ); (**a**) open-aperture results, showing the signature of saturable absorption, and (**b**) closed-aperture z-scan, showing a positive nonlinear refractive index. Dot lines represent experimental data and solid lines correspond to numerical fiting.

To further investigate the ultrafast electronic nonlinearities exhibited by the SiNPs, we then explored the nonlinear optical response of the SiNPs away from resonance and the effect of the N implantation, employing for this purpose, fs pulses at 800 nm. Figure 6a shows typical open-aperture z-scan results obtained for the SiNPs sample, displaying the signature of saturable absorption, i.e., a transmittance maximum at the focal plane. Figure 6b shows the corresponding closed-aperture, where the effect of nonlinear absorption is eliminated by plotting the closed/open-aperture ratio as is customary. The results show the signature of a positive nonlinear refractive index n_2_, i.e., a pre-focal minimum followed by a post-focal maximum. The figure also shows the fit to the data obtained using Equations (3)–(5).

**Figure 7:**
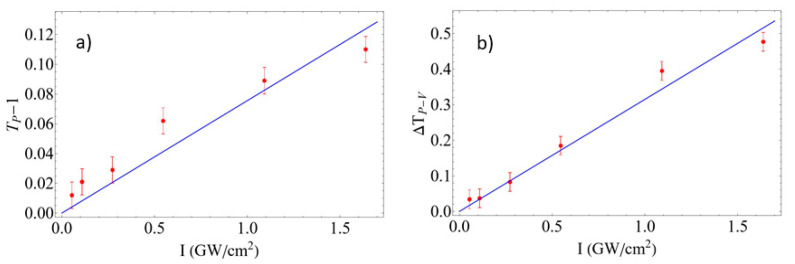
Dependence of the experimental z-scan signals at different input irradiance values *I*_0_ for the SiNPs sample; (**a**) maximum transmittance change *T_p_*−1 for the open-aperture results, and (**b**) peak to valley transmittance change Δ*T_pv_* measured for the closed-aperture z-scan results. In both cases, linear fits to the data are also shown.

Measurements were made at different input irradiance values in order to verify whether we have a purely *χ*^(3)^ nonlinearity or not. For this purpose, in the case of the open-aperture z-scan results, we plot the maximum transmittance change obtained, which we label as *T_p_*−1, the difference between the peak transmittance at the focal plane minus the linear transmittance (obtained far from focus), which is normalized to 1. Figure 7a shows the *T_p_*−1 values extracted from each data set at a given input irradiance *I*_0_, as a function of *I*_0_. The results clearly show a linear relationship, which indicates that we have a purely third-order nonlinearity up to the highest irradiance employed in the experiments. The nonlinear absorption coefficient beta can be extracted from the slope of a linear fit to the data, yielding *β* = −3.97 ± 0.01 × 10^4^ cm/GW. Figure 7b shows the same analysis applied now to the input irradiance dependence of the closed-aperture results. In this case, the plot shows the peak to valley transmittance change *ΔT_pv_* obtained for each *I*_0_ value. The results show again a linear dependence of Δ*T_pv_* with *I_0_* implying a purely *χ*^(3)^ nonlinearity. A fit to the data allows the extraction of the nonlinear refractive index to yield *n_2_* = 0.26 ± 0.01 cm^2^/GW.

For the SiNPs+N sample, no discernable signals were observed either in the open or in the closed-aperture results up to the highest input irradiances available. This indicates that the nonlinear coefficients *β* and *n*_2_ for this sample are considerably smaller than those without N doping. From this, we can only establish an upper bound for the nonlinear coefficients for the SiNPs+N sample, *n*_2_ < 0.071 cm^2^/GW, and *β* < −385 cm/GW. These bounds are obtained considering the coefficients that would produce a transmittance change of 0.01, the minimum discernable signal in the measurements.

## 4. Discussion

The optical bandgap is the minimal energy of the incident photons to promote electrons from the valence band to the conduction band inside the SiNPs. In our samples, the size of the SiNPs is less than 10 nm—short enough to get a spatial confinement to spread the electron wave function in k reciprocal space and overcome the indirect nature of the band structure of Si. The PL peak emission from the SiNPs can be estimated as a function of their average size $d_{0}$ and the variance σ of the nanoparticles size distribution taking into account quantum confinement models. By using the analytical expression [21],

(6) $$PL(\Delta E)=F{\left(\Delta E\right)}^{-k/x}\exp\left\{-(1/2){\left(d_{0}/\sigma \right)}^{2}{\left[{\left(c/(\Delta E\;d_{0}^{x})\right)}^{1/x}-1\right]}^{2}\right\},$$

where *F* is a normalization constant, ΔE is the emission energy, *c* = 4.85816 eV·nm^2^, *x* = 2 and k=r−b+x+1, where *r* is a parameter related to the dimension of the nanoparticles and is equal to 3 for nearly spherical nanoparticles, while *b* is the oscillators strength whose value is taken equal to 5 in our calculations. According to TEM results, the average size of the SiNPs is about 3.5 nm and taking a variance of about 20% the PL peak calculated using Equation (6) is of ~740 nm which corresponds to the PL peaks in Figure 2. However, for embedded SiNPs in SiO_2_ matrices, the presence of surface defects plays an important role that also determines their optical response [22]. These surface defects can be efficiently passivated by the thermal annealing under H or N atoms, and also for the oxygens atoms of the silica matrix. However, these chemical bonding at the surface of the SiNPs introduces electronic states inside the optical band gap. Then even though the optical absorption occurs through the optical band gap (953 to 354 nm) of the SiNPs, the PL emission occurs at lower energies (600 to 850 nm) between electronic states inside the optical band gap [23].

The incorporation of N atoms partially overlapped with the profile depth distribution of SiNPs inside the silica matrix, and it has the effect of modifying the effective optical bandgap of the nanocomposite, as we deduce from the Tauc plots in Figure 2b. The modification of optical bandgap must be an influence over the optical properties, in particular over the third order nonlinear responses. Many strategies have been implemented to engineer the optical bandgap of nanomaterials in order to enhance the Kerr nonlinearity, like in the case of plasmonic materials [24] and silicon nitride films [6]. The lack of any PL signals from the SiNPs at 532 nm excitation indicates that the optical bandgap of the sample is larger than the corresponding photon energy, 2.33 eV, which is consistent with the bandgap values obtained from the Tauc plots (>4 eV). This means that optical nonlinearities in the samples are induced by a multiphoton absorption process that occurs at 532 nm. 

In order to understand the nonlinear response observed and its dependence on different parameters such as light frequency or material bandgap, we can use a two-level model [25]. In this model, the nonlinear susceptibility $\chi^{\left(3\right)}$ can be written as: [26]:

(7) $$\chi^{\left(3\right)}=\frac{4Nm^{4}\left[T_{1}T_{2}^{2}\left(\Delta T_{2}-i\right)\right]}{3\hbar^{3}{\left[1+\Delta T_{2}^{2}\right]}^{2}},\;$$

where Δ = *ω* − *ω*_21_, is the frequency detuning from resonance, where *ω*_21_ = (*E*_2_ − *E*_1_)/*ħ*; *m* corresponds to the atomic dipole moment and the number density of the atoms is represented by *N*. In Equation (4), 1/*T*_1_ designates the population loss through radiative and non-radiative processes of the upper quantum level of excitation, while 1/*T*_2_ refers to the characteristic time of polarization loss. 

For our experiments, nitrogen implantation can be assumed to modify the multiphotonic transitions in the nanocomposites. For the particular case of a large detuning, i.e., Δ*T*_2_ >> 1, the mathematical expression for the magnitude of $\chi^{\left(3\right)}$ is:

(8) $$\left|\chi^{\left(3\right)}\right|\approx \sqrt{{\left(\frac{4}{3}Nm^{4}{\left[\frac{1}{\hbar \Delta }\right]}^{3}\frac{T_{1}}{T_{2}}\right)}^{2}+{\left(\frac{4}{3}Nm^{4}{\left[\frac{1}{\hbar \Delta }\right]}^{3}\frac{T_{1}}{\Delta T_{2}^{2}}\right)}^{2}}.$$

**Figure 8:**
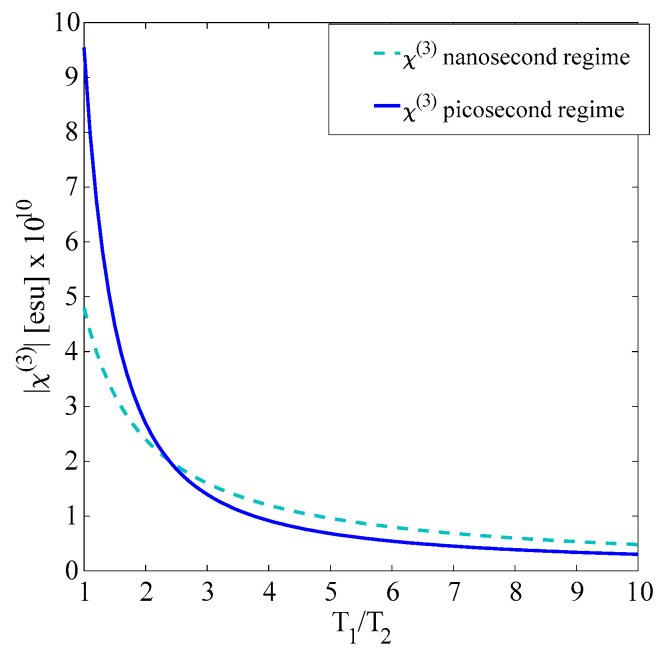
Numerical third-order nonlinear optical susceptibility vs. population loss factor *T*_1_/*T*_2_ exhibited the SiNPs+N sample.

The modification in the bandgap produced by *N* doping can change the resonance conditions through changes in the detuning factor Δ, and eventually, the population losses are suitable to enhance the third-order optical nonlinear properties in different proportions for nanosecond and picosecond pulses. However, for a non-resonant interaction as the studied case, the radiative, spontaneous emission, and the decay given by phonons can be enhanced by the incorporation of defects in the nanocomposites. This off-resonance condition is estimated to be dominant for the change in the nonlinear behavior as a function of the rate of loss factor *T*_1_/*T*_2_ in both ns and ps temporal regimes, as illustrated in Figure 5. It is worth mentioning that the modification in Eg changes the nonlinear response, but the different rates of the loss factor *T*_1_/*T*_2_ for ns and ps pulses can produce results like those exemplified in Figure 8.

Multiphoton absorption is a phenomenon that allows the simultaneous absorption of two or more photons to promote electrons into the conduction band. The probability of this multiphoton absorption process depends on the intensity of the optical field, and the contribution of electromagnetic characteristics of different elements integrated in a substrate must be considered [27]. For the SiNPs, this multiphotonic process is expected to be larger for shorter laser pulses considering that ultrashort interactions can provide a high incident irradiance before reaching the ablation threshold. As we see from the results in Figure 4 and Table 1, the Kerr nonlinearity is larger in the picosecond regime, which is consistent with a dynamical, nonlinear effect involving a multiphoton phenomenon. 

From the off-resonance femtosecond results, we can observe that at 800 nm, a considerably larger nonlinear response is obtained for both the absorptive and refractive contributions for the SiNPs when compared to those for the SiNPs+N sample. This is consistent with the fact that the SiNPs sample shows a stronger linear absorption than that of the sample containing N at this wavelength, which practically has no absorption at 800 nm. The presence of linear absorption in the sample without N implantation is also consistent with the fact that we observed saturable absorption and the refractive nonlinearity associated with it in the femtosecond regime. 

Table 2 shows some recent works with ultra silicon-rich nitride films and their third-order nonlinear optical properties at infrared wavelength excitation. The nonlinear refractive index is of the order of 10^−4^ to 10^−5^ cm^2^/GW, and the two-photon absorption is negligible at this excitation wavelength [14,28,29,30,31]. This is in contrast with the nonlinear optical response observed in our samples, where the nonlinear refractive index is of the order of 10^−2^ to 10^−1^ cm^2^/GW, but the two-photon absorption has a significant value. In order to enhance the $\chi_{eff}$ response of silicon nitride films an approach combining plasmonic nanoparticles made of Ag have been proposed to reach $\chi_{eff}$ values of the order of 10^−7^ esu [32]. In this work, we can also improve $\chi_{eff}$ response of SiNPs by doping with N atoms by ion implantation. At a visible wavelength excitation (532 nm) with picosecond laser the $\chi_{eff}$ for the sample SiNPs+N is three-time enhanced with respect to SiNPs sample in our research, and 5-fold enhanced with respect Si clusters in SiO_2_ synthesized by PECVD methods [33].

Enhanced light-matter interaction in optical nanodevices is highly desired, especially in the development of integrated nonlinear platforms. In particular, the observation of nonlinear effects at lower irradiance is a requisite to implementing all-optical nanosystems. In this work, we have proposed a simple strategy of Si and N co-implantation to enhance the *χ*^(3)^ nonlinear effects by a factor of up to three for the case of picosecond laser irradiation. Consequently, this means that for lower power irradiation, we can obtain a similar nonlinear effect and have the potential to implement this technique in the design of silicon photonics components at the nanoscale with multifunctional operations. 

## 5. Conclusions

PL emission and third-order nonlinear effects from SiNPs embedded in SiO_2_ were studied after a co-implantation with nitrogen ions. The incorporation of N inside the host matrix containing the SiNPs slightly changes the effective optical bandgap of the nanocomposite from 4.5 eV to 4.9 eV. The PL emission from the sample with N increases 27% with respect to the sample with only SiNPs, due to a process of better surface defects passivation. Even though at 532 nm laser excitation, the excitation of the SiNPs by single-photon absorption is not possible, under nanosecond and picosecond excitation, it was possible to observe an enhanced Kerr effect. A three-fold enhancement of the third-order effective susceptibility at 532 nm laser excitation was observed for the sample implanted with N atoms. Femtosecond z-scan evaluations demonstrate a contrast in the influence of the Ni co-implantation over the modification of the ultrafast nonlinearities of the SiNPs samples, resulting in the inhibition of the third-order optical behavior. These findings establish a simple approach for engineering the optical bandgap of nanocomposites, which can be controlled by ion-implanted nitrogen doping. This highlight the potential of enhanced light-matter interactions induced by nitrogen in nanoscale systems. These findings are interesting regarding the attempts to develop nanomaterials with the ability to generate nonlinear effects at lower irradiance and enhance light-matter interactions in nanodevices with multifunctionalities. 

## Figures and Tables

**Table 1:** Effective *χ*^(3)^ susceptibility estimated from the results in Figure 4 for the SiNPs and SiNPs+N samples at 532 nm.

Sample	$\left|\chi_{eff}^{\left(3\right)}\right|$ [esu]	$\left|\chi_{eff}^{\left(3\right)}\right|$ [esu]
	***Irradiation by 4 ns pulses***	***Irradiation by 25 ps pulses***
SiNPs	3.7 × 10^−10^	2.9 × 10^−10^
SiNPs+N	4.81 × 10^−10^	9.28 × 10^−10^

**Table 2:** Third-order nonlinear optical parameters for SiNPs and silicon nitride films.

	$n_{2}$ (cm^2^/GW)	β (cm/GW)	$\chi_{eff}$ (esu)	Platform
Ultra Si-rich Nitride (Si_7_N_3_) [14]	8.1 × 10^−4^ $\left(\lambda_{exc}:\;1000\;\mathrm{nm}\right)$	80 $\left(\lambda_{exc}:\;1000\;\mathrm{nm}\right)$	*-*	PECVD ^1^
Si-rich Nitride and Si-rich oxide layers [28]	2.5 × 10^−5^ $\left(\lambda_{exc}:\;1550\;\mathrm{nm}\right)$	Nil $\left(\lambda_{exc}:\;1550\;\mathrm{nm}\right)$	-	CVD ^2^
Si-rich nitride (Si_7_N_3_) [29]	2.8 × 10^−4^ $\left(\lambda_{exc}:\;1555\;\mathrm{nm}\right)$	Nil $\left(\lambda_{exc}:\;1555\;\mathrm{nm}\right)$	-	CVD
Si-rich Nitride (Si_1.85_N) [30]	1.4 × 10^−5^ $\left(\lambda_{exc}:\;1550\;\mathrm{nm}\right)$	Nil $\left(\lambda_{exc}:\;1550\;\mathrm{nm}\right)$	-	CVD
Si-rich Nitride (Si_1.25_N) [31]	1.6 × 10^−5^ $\left(\lambda_{exc}:\;1550\;\mathrm{nm}\right)$	Nil $\left(\lambda_{exc}:\;1550\;\mathrm{nm}\right)$	-	PECVD
Si clusters in SiO_2_ [33]	~10^−3^ $\left(\lambda_{exc}:\;820\;\mathrm{nm}\right)$	~10^−1^ $\left(\lambda_{exc}:\;820\;\mathrm{nm}\right)$	1.9 × 10^−10^	PECVD
Multilayered Ag: Si_3_N_4_ [32]	-	-	1.1 × 10^−7^	Ion Beam Sputtering
SiNPs in SiO_2_ (This Work)	0.26 $\left(\lambda_{exc}:\;800\;\mathrm{nm},\;\mathrm{fs}\;\mathrm{pulses}\right)$	−3.97 × 10^−4^ $\left(\lambda_{exc}:\;800\;\mathrm{nm},\;\mathrm{fs}\;\mathrm{pulses}\right)$	2.9 × 10^−10^ $\left(\lambda_{exc}:\;532\;\mathrm{nm},\;\mathrm{ps}\;\mathrm{pulses}\right)$	Ion Implantation
SiNPs+N in SiO_2_ (This Work)	≤0.071 $\left(\lambda_{exc}:\;800\;\mathrm{nm},\;\mathrm{fs}\;\mathrm{pulses}\right)$	≤−385 $\left(\lambda_{exc}:\;800\;\mathrm{nm},\;\mathrm{fs}\;\mathrm{pulses}\right)$	9.28 × 10^−10^ $\left(\lambda_{exc}:\;532\;\mathrm{nm},\;\mathrm{ps}\;\mathrm{pulses}\right)$	Ion Implantation

^1^ Plasmon enhanced chemical vapor deposition. ^2^ Chemical vapor deposition.

## Data Availability

Data and materials are available upon reasonable request to C. Torres-Torres (ctorrest@ipn.mx) and J. Bornacelli (jhbornacelli@gmail.com).
